# Association Between Sleep, Beliefs About Sleep, and Executive Functioning in a College Student Sample: The Moderating Role of Dysfunctional Beliefs

**DOI:** 10.3390/healthcare13182373

**Published:** 2025-09-22

**Authors:** Kate Schneider, Gillian Falletta, D. Erik Everhart

**Affiliations:** Department of Psychology, East Carolina University, Greenville, NC 27858, USA; schneiderka22@students.ecu.edu (K.S.); fallettag21@students.ecu.edu (G.F.)

**Keywords:** executive functioning, dysfunctional beliefs about sleep, insomnia severity, sleep duration

## Abstract

**Background/Objectives:** Sleep is integral to cognitive functioning, yet many college students experience poor sleep, often influenced by dysfunctional beliefs about sleep. Dysfunctional beliefs can exacerbate sleep issues and negatively impact executive functioning (EF). Distinct EF facets, including inhibition, working memory, and cognitive flexibility, may differ in their sensitivity to sleep disruptions. While research suggests links between sleep and EF, less is known about how sleep-related beliefs may moderate this relationship and how sleep can affect the various EF facets. Utilizing an undergraduate population, this study examined how sleep quality/quantity affects the different EF facets, how this relationship differs between subjective and objective measurements, and whether dysfunctional beliefs about sleep moderate the relationship. **Methods:** Undergraduate students (N = 212, ages 18–23) completed self-report measures assessing dysfunctional beliefs about sleep (DBAS-16), sleep quality (ISI), and sleep quantity (self-reported sleep duration). Objective EF was measured using computerized CNS Vital Signs tasks targeting inhibition (Stroop Test), working memory (4-Part Continuous Performance Test), and cognitive flexibility (Shifting Attention). Subjective EF was measured using individual subscales on the Behavioral Rating Inventory of Executive Functioning—Adult Version (BRIEF-A). **Results:** Moderation analyses were conducted via linear regression. When measured objectively, neither sleep quantity nor insomnia severity (sleep quality) significantly affected any EF facets, and dysfunctional beliefs about sleep did not have any significant moderation effect. When measured subjectively, insomnia severity (sleep quality), but not sleep quantity, significantly predicted inhibition and cognitive flexibility; in contrast, neither predictor significantly predicted working memory. Regarding specific predictors, dysfunctional sleep beliefs were found to exert significant effect over all three facets; this effect was diminished when insomnia severity was included in the model. Regarding moderation, dysfunctional beliefs about sleep moderated the relationship between sleep quantity and all three EF facets. **Conclusions:** The impact of sleep quality, sleep quantity, and dysfunctional beliefs about sleep varies depending on whether the facets of EF are measured subjectively or objectively. Dysfunctional beliefs about sleep may exacerbate the perceived effect of short sleep duration on daytime cognitive functioning. In addition, insomnia severity may account for the effects of dysfunctional sleep beliefs on perceived inhibitory control and cognitive flexibility; however, working memory may be more resistant to the effects of sleep disturbances and dysfunctional sleep beliefs. Clinical implications of these results and future directions are discussed.

## 1. Introduction

Sleep is an imperative process for humans. Sleep quantity plays a significant role in motor learning, memory formation, and neurocognitive performance [1]. Most teenagers and adults require 7–9 h of sleep per night [2]. However, Gaultney [3] found that college students average approximately 6.79 h of sleep on a typical work/school night.

In addition to low sleep quantity, Humphries et al. [4] found that approximately 60% of college students endorse poor sleep quality. An abundance of studies have emphasized a relationship between inadequate sleep hygiene (i.e., caffeine consumption, daytime napping, etc.) and poor sleep quality among college students [5,6,7]. Consequently, students are at risk of experiencing symptoms such as increased tension, irritability, depression, confusion, and impaired learning [8,9]. College students ranked sleep difficulties as the third leading health impediment to academic performance [10].

### 1.1. Beliefs About Sleep

Beliefs about sleep refer to subjective expectations and perceptions about recent sleep and sleep patterns, including beliefs about how much sleep is required, how optimal recent sleep has been, and the consequences of inadequate sleep. Beliefs about sleep are more subjective than other measures; however, they have a significant impact on functional abilities [11]. Indeed, the cognitive model of insomnia elucidates the significant relationships between cognitive processes and sleep difficulties. This model, initially proposed by Harvey [12], illustrates how excessive, negative cognitive activity (e.g., persistent worry about sleep and the daytime consequences of not getting enough) can trigger autonomic arousal and emotional distress which, in turn, can activate a state of hyperfocus on counterproductive behaviors (e.g., clock watching, catastrophizing, etc.) that then contribute to sleep disturbances. Specifically, dysfunctional beliefs about sleep may disrupt sleep at night. College students experiencing poor sleep have an increased likelihood of engaging in dysfunctional sleep hygiene practices including daytime napping, caffeine consumption, and having a noise-filled bedroom environment [13,14]; these behaviors tend to be counter-productive, often perpetuating sleep disturbances.

Moreover, although individuals who subscribe to dysfunctional beliefs about sleep tend to overstate perceived daytime deficits, the excessive and persistent anxiety, paired with subsequent sleep disruption, may culminate into functional deficits [12]. For example, Semler & Harvey [15] found that college students who received negative feedback about their sleep exhibited more negative thoughts (i.e., “I’m never going to cope today”), higher levels of sleepiness, increased monitoring for sleep-related threats (i.e., headaches, backaches), higher use of safety behaviors (i.e., canceling nighttime plans to accommodate for excess time in bed) than when they received positive feedback; this was despite the fact that sleep quantity remained consistent [15]. Similarly, a study by Rahman et al. [16] found that participants processing speed was significantly slower when participants slept 8 h but were told they slept 5 h and was significantly higher when they slept 5 h but were told they slept 8 h; this indicates that perceived sleep quantity may have a stronger impact on daytime cognitive abilities than objective sleep quantity [16].

There is also evidence that participants with dysfunctional beliefs about sleep may perform worse on neuropsychological tests. Fortier-Brochu and Morin [11] found that individuals with insomnia attained lower scores on neuropsychological measures than matched controls; however, notably, individuals with insomnia who had a higher frequency of complaints regarding the effects of insomnia on cognitive abilities had more impaired performance than those with less complaints [11].

Despite the literature introduced thus far underlining the significant effect of cognition on daytime functioning, other studies yield opposing conclusions. For example, Zavecz et al. [17] examined data from three studies utilizing a healthy undergraduate population. The results showed no significant association between subjective sleep disturbance and objective performance in the cognitive domains of EF, working memory, processing speed, and procedural learning [17].

### 1.2. Executive Functioning (EF)

EF is one of the areas of cognitive functioning most negatively affected by poor sleep. This neuropsychological construct can be difficult to operationally define, as it is multifaceted and consists of a variety of interacting higher-order cognitive processes. EF is necessary for performing any novel and/or complex task for which an automatic, routine response does not suffice and can be defined as a culmination of processes that include [18] (p. 106) the following:

(1) Forming, (2) maintaining, and (3) shifting mental sets, corresponding to the abilities to (1) reason and generate goals and plans, (2) maintain focus and motivation to follow through with goals and plans, and (3) flexibly alter goals and plans in response to changing contingencies (p. 106).

Although a multitude of cortical and subcortical regions are involved in the cognitive processes encompassed by the construct of EF, the prefrontal cortex plays a dominant role, and this brain region is sensitive to sleep loss [19]. Throughout recent years, studies note a link between sleep disturbances and EF abilities [20,21], and some studies found that subjective reports about sleep, as opposed to objective sleep data (i.e., sleep quantity, etc.), more largely affect neurocognitive skills [22].

Tasks designed to measure EF as a whole can fall victim to a “task impurity problem” [19], ultimately measuring a variety of cognitive processes instead of one particular ability, which can lead to performance misattribution. While frequently regarded as one cognitive domain, research supports that EF comprises multiple, distinctive components that, while related, serve more specialized functions [23]. The three main components consist of inhibition (i.e., the ability to control impulsive, automatic responses), working memory (i.e., the ability to apprehend and hold information in immediate awareness, manipulate it, and produce a desired result), and cognitive flexibility (i.e., ability to shift attention between different tasks and behaviors) [23]. Poor performance on an EF task may lead a clinician or researcher to assume a more global functional impairment; however, as the various facets of EF are differentially impacted by sleep loss, such impairment may be limited to one specific facet [24].

Regarding the cognitive component of inhibition, research has yielded mixed findings when analyzing the association between lower sleep quantity and tasks measuring inhibitory control. Two studies found that adults with insomnia Fortier-Brochu and Morin [11] and those who experienced 24-h sleep deprivation (Magnuson et al. [25]) performed significant worse on tests of inhibition than their counterparts without insomnia or who obtained adequate sleep, respectively. However, a larger amount of literature has reported no significant association between sleep difficulties and reduced inhibitory control [26,27]. Examining the EF component of working memory and sleep quantity, the literature has offered more consistency. A study by Cellini et al. [28] concluded that “primary insomniacs” exhibit more impairments than “good sleepers” when engaging in tasks involving greater cognitive load on working memory but did not exhibit such detriments when engaging in simpler tasks that require lower cognitive load. Similarly, Shekleton et al. [29] found that adult insomnia patients performed significantly worse on working memory tasks than healthy controls but showed no differences in performance on simple or complex sustained attention tasks. Regarding individuals without a clinical sleep diagnosis who underwent 36 h of sleep deprivation, younger individuals (ranging from 19 to 38 years) experienced significantly larger working memory dysfunction than older individuals (ranging from 59 to 82 years) [30]. Finally, regarding the component of cognitive flexibility, the majority of the literature finds no significant group differences between individuals with insomnia and those attaining normal sleep quantity [27,31,32,33,34].

To consider the complexities that accompany the relationship between sleep and cognitive performance, it is beneficial to review a study assessing potential moderators of this relationship. The meta-analytic review conducted by Lowe et al. [35] evaluated multiple variables that could serve as potential moderators regarding the effect of sleep restriction on neurocognitive functioning. Analyses revealed that age-adjusted sleep deficit and cumulative days of restricted sleep moderated the relationship between sleep restriction and overall cognitive functioning; however, they were not significant moderators of the effect of sleep restriction specifically on EF, especially regarding its subcomponents of working memory and inhibition [35]. This suggests that EF is particularly sensitive to the effects of inadequate sleep, as deficits are detectable regardless of severity and constancy.

### 1.3. The Present Study

A multitude of studies have analyzed the effects of sleep on EF [25,28,30]. Similarly, studies have also assessed the relationship between dysfunctional beliefs about sleep and factors such as sleep quality and daytime dysfunction [12,36]. However, to our knowledge, no studies have looked at the relationships among all three of these factors. As a result, the present study sought to examine the relationships between sleep (including sleep quantity and insomnia severity), cognitions about sleep, and the three facets of EF in college students. Additionally, this study explored the potential moderating relationship of dysfunctional beliefs about sleep on the relationship between sleep and EF. While conducted as an exploratory analysis, we hypothesized that the three facets of EF would be differentially impacted by the aforementioned variables, as well as the moderation, and that such impact would differ between objective measures and subjective measures. Understanding the role of perceptions and beliefs about sleep may allow clinicians to intervene and mitigate executive dysfunction in novel ways. Such interventions will be especially beneficial for individuals who experience executive dysfunction but, due to extraneous factors (i.e., hectic schedule, less developed time-management skills, etc.), are not able to adjust their sleep quantity to the recommended level. College students are a key example of a population who may fall under the purview of this issue.

## 2. Materials and Methods

### 2.1. Participants and Procedures

This study was approved by the University Medical Center Internal Review Board (UMCIRB #24-001655) at East Carolina University. Study participants (*n* = 212) comprised undergraduate students enrolled in introductory psychology courses and were recruited through the SONA online research participation platform. Eligibility criteria included being between the ages of 18–23 years and proficiency in the English language. As compensation for study completion, participants received course credit that toward fulfillment of their research activity requirement for an introductory psychology course. See Table 1 for demographic characteristics of the participants.

Participants completed all study surveys through a REDCAp platform. After REDCAp survey completion, participants accessed an emailed link to the CNSVS website to complete EF-related measures. Each component was completed online using a desktop or laptop computer.

### 2.2. Measures and Questionnaires

#### 2.2.1. Sleep Questionnaires

The Insomnia Severity Index (ISI) was selected to measure subjective sleep quality. It is a brief self-report questionnaire designed to assess qualities including one’s satisfaction with sleep patterns, the severity of an individual’s insomnia, and its subsequent impact on the individual’s daily functioning over the past two weeks. Scores for all seven items are summed to produce one total score, with higher scores indicating more severe insomnia. The ISI has been deemed reliable and valid regarding the detection of insomnia [37] and analysis of its psychometric properties illustrates internal consistency (Cronbach’s alpha = 0.74) [38].

Additionally, the abbreviated version of Dysfunctional Beliefs and Attitudes about Sleep Scale (DBAS-16) is a 16-item self-report measure designed to assess an individual’s subjective cognitions (including beliefs, attitudes, expectations, etc.) about sleep. A composite score is attained by calculating the mean of the responses, with higher means indicating more unrealistic expectations for sleep and/or thoughts about sleep have become a factor in the individual’s sleeping difficulties. Adequate construct validity regarding this measure is reflected via its factor structure aligning with contemporary conceptualizations of insomnia, and adequate internal consistency has been noted (Cronbach’s alpha = 0.77) [39].

Lastly, the Pittsburgh Sleep Quality Index (PSQI) was utilized to attain participant sleep quantity. The PSQI is a self-report questionnaire designed to assess an individual’s quality of sleep over a one-month time interval. It comprises 19 items that bridge seven clinical domains including subjective sleep quality, sleep latency, sleep duration, sleep efficiency, sleep disturbances, use of sleeping medications, and daytime dysfunction. For this study, only item 4 (i.e., During the past month, how many hours of actual sleep did you get at night?) was used in the final analyses and was operationally defined as “sleep duration.” Analysis of this measure’s psychometric properties illustrates good validity [40], internal consistency (Cronbach’s alpha = 0.83), and test–retest reliability (*r* = 0.85) [40].

#### 2.2.2. Subjective EF Measures

The Behavioral Rating Inventory of Executive Function for Adults (BRIEF-A) is a standardized self-report measure designed to assess executive function abilities in an individual’s everyday environment. It comprises 75 items that bridge nine empirically derived clinical scales including (1) inhibit, (2) self-monitor, (3) plan/organize, (4) shift, (5) initiate, (6) task monitor, (7) emotional control, (8) working memory, and (9) organization of materials. This measure has demonstrated evidence of good test–retest reliability (*r* = 0.82–0.94) and moderate to high internal consistency (Cronbach’s alpha = 0.73–0.90) [41].

#### 2.2.3. Objective EF Measures

CNS Vital Signs (CNSVS): This is a web-based cognitive screening battery designed to detect mild cognitive dysfunction. It was used to assess various facets of EF via a custom test battery comprised for the present study. Upon battery completion, CNSVS auto-scores participant responses and provides summary information including raw scores, standard scores, percentiles, qualitative classifications, and validity indicators. Test scores are age-adjusted and were normed utilizing 1508 individuals ages 8–90 who were seen by a provider and deemed “healthy” (i.e., no active, uncontrolled medical conditions and no history of neurological or psychiatric disorders). The CNSVS test battery included:

Stroop Test (ST): This test is designed to measure an individual’s reaction time and inhibition abilities [42]. The CNSVS Stroop Test is adapted from the original Stroop Test [43]; in this version, the words are presented on a computer screen, and participants press the space bar to signal their responses. The CNSVS Stroop Test contains three parts. Part one presents the words RED, YELLOW, BLUE, AND GREEN as printed in black ink; participants were instructed to press the spacebar as soon as they see the word. Part two presented the words printed in various ink colors; participants were instructed to press the space bar when the ink color was congruent with the presented word. For part three, participants were instructed to press the space bar when the color was not congruent with the presented word. Test scores related to reaction time and commission errors were calculated, with higher standard scores corresponding with more developed inhibition abilities.

Shifting Attention (SAT): This test measures an individual’s ability to effectively and efficiently shift from one instruction to another, which exemplifies cognitive flexibility. In this test, participants saw three geometric objects (either squares or circles), with one on top and two on the bottom. These objects were colored either red or blue. Participants were tasked with matching one of the bottom objects to the top object, either by color or by shape, with the rule changing at random; participants had two seconds to respond. Test scores related to participants’ number of correct matches, number of errors, and response time are calculated, Test scores related to participants’ number of correct matches, number of errors, and response time are calculated, with higher standard scores corresponding with more developed cognitive flexibility abilities.

4-Part Continuous Performance (FPCPT): This four-part test is designed to measure an individual’s working memory and sustained attention abilities. Part one instructs participants to press the space bar when any shape appears on the screen; it tests an individual’s simple reaction time. Part two specifies that participants only press the space bar when they see a certain colored shape. Part three instructs participants to press the space bar when the currently presented figure is the same as the figure immediately preceding it (i.e., a “one back” continuous performance test [CPT]). Part four instructs participants to press the space bar when they see two objects that are identical in shape and color but are separated by a different object (i.e., a “two-back” CPT). Test scores related to participants’ number of correct responses, number of omission errors, and response time are calculated for each of the four parts of the tests; higher standard scores correspond with more developed working memory abilities.

### 2.3. Validity Checks

Validity items consisting of unusual statements (i.e., “how often have you talked with a street performer about the best way to juggle flaming torches?”) were embedded within the ISI and DBAS-16, which did not originally include validity checks. The BRIEF-A already included three embedded validity scales (i.e., Inconsistency, Negativity, and Infrequency) to identify if participants responded to the assessment in a consistently appropriate fashion. Neuropsychological tests conducted via CNSVS all contained embedded validity measures (see Appendix A.1). Validity criteria were automatically evaluated and interpreted by CNSVS. Participants were excluded from the present study if they failed two or more validity measures across the comprehensive test battery.

### 2.4. Data Analysis Plan

Only participants who completed all components of each self-report measure and neurocognitive test were included in the analyses; participants with missing data were excluded. A total of 1006 participants completed the self-report measures and were subsequently emailed a link to the neurocognitive tests; 268 individuals completed this portion, thereby finishing the study.

Several validity checks were embedded throughout the assessments. For the ISI and DBAS-16, manual review of validity questions ensured responses were appropriate; BRIEF-A responses were considered invalid if any validity scale exceeded its cutoff; and neurocognitive test data were flagged as invalid if CNSVS identified any domain as such. Participants failing two or more validity checks were excluded. After applying these criteria, 212 participants met all validity requirements and had complete data, and were therefore included in the present analyses.

Twelve exploratory moderation analyses were performed to assess the relationship between the different facets of EF (inhibition, working memory, and cognitive flexibility) and predictor variables of sleep quantity, insomnia severity, and dysfunctional beliefs about sleep, with dysfunctional beliefs about sleep also moderating these relationships. Age and sex were controlled for in each model.

## 3. Results

Statistical analyses were conducted using the statistical computing environment R (Version 2023.03.0 + 386, R Core Team, 2022). All but one of the 212 participants included in the study reported their age; the mean age was 18.42 years (SD = 0.80). See Table 1 for additional demographic information.

Though not included in present analyses, there are beneficial significant correlations to observe; see Table 2 below.

The means and standard deviations were calculated for the key measures utilized in this analysis (Appendix A.2). Of note, the mean sleep quantity for study participants, as attained from responses on the PSQI, was 7.00 h (SD = 1.36). This indicates that, on average, participants in this sample attained slightly more sleep per night than the typical college student, as Gaultney [3] found the average sleep quantity to be 6.79 h of sleep per night. However, this amount of sleep is still significantly below the recommended amount of sleep (i.e., 9.5 h) for teenagers per the National Institute of Neurological Disorders and Stroke [2]. Further, it can be observed that both the mean CNSVS EF score (SS = 92.97) and BRIEF-A GEC score (T = 54.01) are within the average range of functioning. CNSVS scores are slightly lower than the population mean (SS = 100), and BRIEF-A GEC scores are slightly higher than the population mean (T = 50); however, both are still within one standard deviation of this mean, which suggests that the attained sample is an adequate representation of the population with respect to EF. One can also note that the mean score on the ISI was 7.76 (SD = 5.42). This indicates that, on average, participants’ scores fell between no clinically significant insomnia (score range is 0–7) and subthreshold insomnia (score range is 8–14).

### 3.1. Objective Measures of Inhibition, Cognitive Flexibility, and Working Memory

To assess the facets of EF through objective measures, regression models utilizing CNSVS scores regarding Stroop Commission Errors were constructed to assess inhibition; models utilizing CNSVS scores regarding the Shifting Attention Test Correct Responses were constructed to assess cognitive flexibility; models utilizing CNSVS scores regarding 4-Part CPT: Part 4 Correct Responses were constructed to assess working memory. Assumptions of multiple linear regression, including linearity, independence, normality, and homoscedasticity as they relate to the model’s residuals (errors) were assessed. Although the assumption of normality was not initially met within the models assessing inhibition and working memory, transformations and the Central Limit Theorem were applied and allowed for parametric analyses to still be utilized with validity.

Both regression models regarding objective inhibition yielded significant results (sleep quantity model: (*F*(6, 204) = 4.83, *p* < 0.001); insomnia severity model: (*F*(6, 204) = 4.69, *p* < 0.001)), accounting for approximately 12% of the variance in objective inhibition scores. One predictor variable, age, was found to significantly influence inhibition scores; a one-year increase in an individual’s age was associated with an average decrease of approximately 5 units in the individual’s CNSVS inhibition score. No additional regression models assessing objective facets of EF were significant; no predictor variables or moderations were significant. See Table 3 below for the full statistical results.

### 3.2. Subjective Measure of Inhibition

When assessing subjective inhibition, a regression model utilizing BRIEF-A inhibition scores was constructed. Assumptions of multiple linear regression were met. The model attained statistical significance (*F*(6, 204) = 5.49, *p* < 0.001); included predictors accounted for 13.9% of the variance in participants’ BRIEF-A inhibition scores. Dysfunctional beliefs about sleep were identified as significant; when controlling for age, sex, sleep quantity, a one-unit increase in an individual’s DBAS score (i.e., their level of dysfunctional beliefs about sleep) was associated with an average increase of 4.90 (*p* < 0.01, 95% *CI* [2.62, 7.18]) in the individual’s BRIEF-A inhibition score. The model’s moderation term also attained high significance, indicating that the effect of sleep quantity on one’s BRIEF-A inhibition score depends on the value of their DBAS score.

Another regression model was performed, substituting sleep quantity with insomnia severity as a predictor. Assumptions of multiple linear regression were met. This model was also statistically significant (*F*(6, 204) = 8.53, *p* < 0.001), with the predictors collectively accounting for 20.05% of the variance in BRIEF-A inhibition scores. The variable of insomnia severity (ISI score was identified as a significant predictor; when controlling for sex, age, and dysfunctional beliefs about sleep, a one-unit increase in an individual’s insomnia severity is associated with an average increase of 0.83 (*p*< 0.05, 95% *CI* [0.11, 1.56]) units in the individual’s BRIEF-A inhibition score. Dysfunctional beliefs about sleep did *not* significantly moderate the relationship between insomnia severity and BRIEF-A inhibition scores. See Table 4 below for the full statistical results.

### 3.3. Subjective Measure of Cognitive Flexibility

When assessing cognitive flexibility through subjective means, a regression model utilizing BRIEF-A shift scores was constructed. Assumptions of multiple linear regression were met. The model attained statistical significance (*F*(6, 204) = 4.95, *p* < 0.001); included predictors accounted for 12.70% of the variance in participants’ BRIEF-A shift scores. Dysfunctional beliefs about sleep were identified as significant; when controlling for age, sex, sleep quantity, a one-unit increase in an individual’s DBAS score (i.e., their level of dysfunctional beliefs about sleep) was associated with an average increase of 4.91 (*p* < 0.001, 95% *CI* [2.41, 7.42]) in the individual’s BRIEF-A shift score. The model’s moderation term was also found to have high significance; when controlling for age and sex, for each unit increase in an individual’s DBAS score (i.e., their level of dysfunctional beliefs), the effect of sleep quantity on a participant’s BRIEF-A shift score decreases by 0.42 (*p*< 0.001, 95% *CI* [−0.64, −0.21]) units.

Another regression model was performed, substituting sleep quantity with insomnia severity as a predictor. Assumptions of multiple linear regression were met. This model was also statistically significant (*F*(6, 204) = 7.90, *p* < 0.001), with the predictors collectively accounting for 18.85% of the variance in BRIEF-A shift scores. The variable of insomnia severity (ISI score) was identified as a significant predictor; when controlling for sex, age, and dysfunctional beliefs about sleep, a one-unit increase in an individual’s insomnia severity is associated with an average increase of 1.06 (*p*< 0.01, 95% *CI* [0.27, 1.86]) units in the individual’s BRIEF-A shift score. Dysfunctional beliefs about sleep did not significantly moderate the relationship between insomnia severity and BRIEF-A inhibition scores. See Table 4 above for the full statistical results.

### 3.4. Subjective Measure of Working Memory

When assessing subjective working memory, a regression model utilizing BRIEF-A working memory scores was constructed. Assumptions of multiple linear regression were met. The model attained statistical significance (*F*(6, 204) = 8.81, *p* < 0.001); included predictors accounted for 20.58% of the variance in participants’ BRIEF-A working memory scores. Dysfunctional beliefs about sleep were identified as significant; when controlling for age, sex, sleep quantity, a one-unit increase in an individual’s DBAS score (i.e., their level of dysfunctional beliefs about sleep) was associated with an average increase of 6.53 (*p* < 0.001, 95% *CI* [3.74, 9.31]) in the individual’s BRIEF-A working memory score. The model’s moderation term was also found to have high significance; when controlling for age and sex, for each unit increase in an individual’s DBAS score (i.e., their level of dysfunctional beliefs), the effect of sleep quantity on a participant’s BRIEF-A working memory score decreases by 0.53 (*p* < 0.001, 95% *CI* [−0.77, −0.29]) units.

Another regression model was performed, substituting sleep quantity with insomnia severity as a predictor. Assumptions of multiple linear regression were met. This model was also statistically significant (*F*(6, 204) = 11.83, *p* < 0.001), with the predictors collectively accounting for 15.81% of the variance in BRIEF-A working memory scores. Male sex was identified as a significant predictor; when controlling for age, insomnia severity, and dysfunctional beliefs about sleep, identifying as a male is associated with an average decrease of 5.18 (*p* < 0.01, 95% *CI* [−9.05, −1.32]) units in the individual’s BRIEF-A working memory score. Dysfunctional beliefs about sleep did not significantly moderate the relationship between insomnia severity and BRIEF-A working memory scores.

## 4. Discussion

### 4.1. Summary of Results and Relevant Implications

The goal of this study was to examine the relationship between sleep quality/quantity, dysfunctional beliefs about sleep, and the three facets of EF in an undergraduate student sample. Prior research has detailed how poor sleep can negatively affect daily functioning and academic achievement [4,13,14]. Similarly, neurocognitive literature has also highlighted the effects of insufficient sleep on EF. However, when dividing this multifaceted cognitive domain into different components, research has shown mixed results, with inhibition, working memory, and cognitive flexibility impacted by sleep in a unique way. The present study sought to provide more clarity regarding the impact of sleep on these distinct facets of EF. Additionally, as negative cognitions about sleep affect sleep itself, as well as daytime functioning and cognitive performance, the current study also sought to bridge the gap in the literature and assess how dysfunctional beliefs (i.e., sleep related cognitions) about sleep may moderate the relationship between sleep and the distinct facets of EF using objective (i.e., neuropsychological testing) and subjective (i.e., self-report) measures.

### 4.2. Objective Measures of EF

This study found no significant relationships between sleep quantity nor sleep quality (insomnia severity) and the objective measures of EF (i.e., inhibition, cognitive flexibility, and working memory). The moderation terms for each model were also non-significant. With regard to inhibition and cognitive flexibility, these results align with a large body of literature that reports non-significant associations between these variables [26,27,31,34]. However, these results are inconsistent with a modest body of literature that identified working memory impairments in relation to decreased sleep quantity [28,29,30]. Yet, similar to the current study, two studies [44,45] also used n-back tasks and failed to find significant associations between sleep and working memory. This may suggest that sleep has a stronger effect on certain types of working memory processes not captured by the n-back task. For example, the n-back task primarily measures the continuous updating and maintenance of information in working memory, as well as interference resolution [46]. However, other working memory tasks like spatial span and letter-number sequencing primarily assess one’s storage capacity, manipulation, and retrieval processes [47]. Sleep may impact one’s latter abilities more than the former.

### 4.3. Subjective Measures of EF

In contrast to the findings when objective measures of EF were utilized, sleep quantity and insomnia severity both showed significant associations with the facets of EF when measured subjectively.

#### 4.3.1. Inhibition

The moderation analysis with respect to subjective inhibition abilities yielded varied results. Sleep quantity was not associated with subjective inhibition score. In a second linear regression model in which sleep quantity was replaced by insomnia severity, insomnia severity was identified as the only predictor that significantly predicted subjective inhibition. These results suggest that perception of inhibition abilities may be more sensitive to sleep quality than total sleep quantity. Further, dysfunctional cognitions about sleep were significant in the model that included sleep quantity, but not significant in the model that included insomnia severity. This suggests that insomnia severity may account for the effects of such cognitions, as embodied in the cognitive model of insomnia [12]. Interestingly, the results support a significant moderation regarding the interaction of sleep quantity and dysfunctional cognitions about sleep, but no significant moderation regarding the interaction of insomnia severity and dysfunctional cognitions about sleep. Taken together, this indicates that individuals with higher levels of dysfunctional cognitions about sleep may be more sensitive to variations in sleep quantity and may perceive even minor reductions in sleep as harmful to their inhibition abilities. However, individuals with lower dysfunctional cognitions about sleep may be more resilient to changes in sleep quantity.

#### 4.3.2. Cognitive Flexibility

When conducting the moderation analysis on subjective cognitive flexibility abilities, the results paralleled the findings with subjective inhibition. Insomnia severity was significantly associated with subjective cognitive flexibility, but sleep quantity was not, suggesting that perceived cognitive flexibility may be more influenced by sleep quality than by sleep quantity. Additionally, dysfunctional beliefs about sleep only emerged as a significant predictor in the model with sleep quantity and not in the model with insomnia severity, again indicating that insomnia severity explains much of the impact attributed to dysfunctional beliefs. Finally, a significant moderation effect for the interaction between sleep quantity and dysfunctional beliefs about sleep was observed, whereas the interaction between insomnia severity and dysfunctional beliefs about sleep was non-significant. These findings suggest that individuals with stronger dysfunctional beliefs about sleep may be more sensitive to fluctuations in sleep quantity, and those with lower levels of dysfunctional beliefs about sleep may demonstrate greater resilience to changes in sleep quantity. It is reasonable that individuals who perceive themselves as having difficulty with inhibiting impulsive responses may also perceive themselves to struggle with shifting their thinking and adapting to new situations, and that these perceptions may be further influenced by sleep.

#### 4.3.3. Working Memory

The moderation analysis of subjective working memory abilities yielded some novel results. Similar to the findings attained for subjective inhibition and cognitive flexibility, no significant relationship was found between sleep quantity and subjective working memory. However, in contrast, insomnia severity was not identified as a significant predictor of subjective working memory scores. It is possible that perceived working memory abilities are more resilient to the effects of impaired sleep quality than perceived inhibitory control or cognitive flexibility. However, it could also be true that working memory complaints are less pronounced because participants may not perceive working memory lapses as readily as they notice difficulties with inhibition (impulse control) and cognitive flexibility (adapting to change). Moderation effects suggest a significant effect for the interaction between sleep quantity and dysfunctional beliefs about sleep and no significant effect for the interaction between insomnia severity and dysfunctional beliefs about sleep, again aligning with the findings from subjective inhibition and cognitive flexibility. These findings convey that individuals with stronger dysfunctional beliefs about sleep may be more vulnerable to changes in sleep quantity, whereas those with lower levels of dysfunctional beliefs about sleep may exhibit greater resilience to sleep fluctuations and may not perceive such changes as detrimental to their working memory abilities.

### 4.4. Limitations and Future Directions

Limitations of this study should be considered. First, as cross-sectional data were utilized, alternative explanations for the findings cannot be ruled out. Specifically, without establishing the temporal order of the variables, causality cannot be inferred, and conclusions must be limited to associations between variables, rather than directional effects.

It should also be acknowledged that the BRIEF-A inhibition, shift, and working memory scores have a moderate to strong, statistically significant correlation with each other (*r* = 0.66–0.69, *p* < 0.01). This means that such perceived abilities are not fully independent constructs, at least to the extent at which they are measured in the BRIEF-A. However, these associations do not negate the interpretations that can come about from this pattern of results. It is reasonable that individuals who perceive themselves as having difficulty with inhibiting impulsive responses may also perceive themselves to struggle with shifting their thinking and adapting to new situations, and that these perceptions may be intertwined when they regard how they are affected by sleep.

Next, the sample utilized in this study had a narrow age range (18–23 years old). Although this age range is characteristic of the “typical” undergraduate population, it may not be expansive enough to detect age differences in EF abilities. Further, the majority of participants (91.98%) were 18–19 years old and over half (50.94%) were in their first semester of college. This may introduce selection bias, as first-year students may differ from upperclassman in their sleep patterns, stress levels, and EF abilities, given the unique challenges of transitioning to college life. In addition, over three quarters of the sample (76.42%) identified as female. The overall undergraduate enrollment in the U.S., as well as at ECU, is approximately 58% female [48,49], and the sample attained for this study significantly surpasses this statistic. However, consistent with our findings, the National Science Foundation, National Center for Science and Engineering Statistics [50] has estimated that 78% of psychology undergraduates identify as female. While not all students in introductory psychology courses are psychology majors, this statistic helps contextualize the female-skewed composition of our sample. This current sample is not veritably representative of the national undergraduate student population, which limits the generalizability of the study findings.

In addition,, online self-reports measures were utilized; although validity questions were incorporated, they do not ensure that participants fully engaged with or accurately responded to each item. Future research may benefit from conducting the study in-person with direct oversight from a researcher so that responses can be more closely monitored.

Further, while confounding variables including age and sex were controlled for, there are a multitude of other variables that could impact an individual’s EF abilities and sleep. These variables include anxiety, depression, and ADHD, the use of certain drugs (i.e., alcohol, cannabis, etc.), and chronic illnesses. Specifically, ADHD symptoms are associated with increased prevalence of insomnia and sleep disturbances [51]. Cifre et al. [52] conducted a moderation analysis regarding the effect of sleep on neurocognitive abilities in an undergraduate population. Results showed that factors including state sleepiness (defined as a longstanding drive to seek sleep), sleep debt (defined as lacking adequate rest), and ADHD symptomatology (specifically, high impulsivity) moderated this relationship. As a result, it is possible that the inclusion of these additional variables could have also increased the proportion of variance accounted for in attained executive functioning scores. Future studies are encouraged to engage in more thorough data collection processes including assessing for underlying psychological conditions, as well as utilizing objective sleep measures (e.g., actigraphy) so that additional potential confounding variables can be accounted for. It is also acknowledged that Actigraphy is considered the “gold standard” when used as an objective measure of sleep. Though utilization of this measure was not feasible in this study due to constraints, it represents a limitation.

Recent studies have observed a U-shaped relationship between self-reported sleep quantity and executive function, indicating that performance tends to decline with both shorter and longer sleep quantity, with optimal functioning around 7–8 h of sleep [53]. However, the present study exclusively focused on the effects of insufficient sleep (i.e., shorter sleep quantity) due to college student reports of lower average sleep quantity [3,4]. Future research should consider whether the relationship between sleep quantity and executive function differs when longer sleep quantity is also considered. In the future, a longitudinal study that incorporates Cognitive Behavioral Therapy for Insomnia (CBT-I) may assist with determination of causality.

## 5. Conclusions

The goal of this study was to examine the complex relationships between sleep, dysfunctional beliefs about sleep, and the three facets of EF (inhibition, cognitive flexibility, and working memory) in a college student sample. When the facets of EF were measured objectively, the results illustrated no significant relationships between them and either variable of sleep quantity (sleep quantity) or sleep quality (insomnia severity). Similarly, in moderation analyses, dysfunctional beliefs about sleep did not exhibit a moderating effect.

When the facets of EF were measured subjectively, sleep quantity was not found to significantly impact any facet. In fact, the primary factor found to exert its effect over these three domains was dysfunctional beliefs about sleep. However, when insomnia severity was added as a predictor, the impact of dysfunctional beliefs about sleep (as well as its moderation effect) was found to dissipate. This provides evidence that insomnia severity accounts for much of the variance attributed to dysfunctional beliefs about sleep and has a stronger impact on perceived EF as a whole, as well as one’s perceived inhibitory control and cognitive flexibility. Yet, it is important to note that insomnia severity did not emerge as a significant predictor of subjective working memory scores. This suggests that perceived working memory abilities may be more resistant to the effects of poor sleep quality, as well as the effects of dysfunctional beliefs about sleep, compared to inhibitory control and cognitive flexibility.

### Clinical Implications

This study begets important clinical implications. First, the lack of a significant relationship between sleep variables and objective facets of EF measures suggests that perceived cognitive impairments may not always reflect veritable, measurable deficits. As such, clinicians should be cautious when interpreting self-reported cognitive difficulties and should consider objective cognitive assessments before attributing executive dysfunction solely to sleep issues. However, it is important to note that, although no objective deficits may be noted in relation to sleep, this does not mitigate the distress individuals may experience regarding their perceived impairments. This notion engenders encouragement for clinicians to provide psychoeducation to patients regarding the presence of perceptual distortions in self-reported impairments. This discussion may lend itself to subsequent treatments that help the patient to challenge their perceptual distortions which, in turn, may help reduce negative sleep-related cognitions and subsequently prevent self-fulfilling sleep and cognitive concerns.

Further, as dysfunctional beliefs about sleep were found to moderate the relationship between sleep quantity and subjective facets of EF, this indicates that individuals who hold strong maladaptive beliefs about sleep may be more prone to perceiving executive dysfunction. This finding is important, as it allows clinicians to better conceptualize a patient’s presented complaints by providing empirical evidence that links these variables together. Subsequently, it allows clinicians to better tailor their treatments to mitigate these difficulties. For example, if a patient is presenting with extensive, dysfunctional beliefs about sleep, engaging them in cognitive behavioral therapy for insomnia (CBT-I) may be beneficial not only in challenging and restructuring these dysfunctional beliefs, but will also likely have benefits that extend to their perceived cognitive functioning.

Also, the finding that insomnia severity, rather than sleep quantity, predicts perceived EF highlights the importance of addressing sleep quality over mere sleep quantity. Although sleep quality has been labeled as a more optimal index for assessing sleep, sleep quantity is often easier to measure and, subsequently, it is easier to become preoccupied with getting “enough” sleep [54]. For example, a common counterproductive behavior for individuals with high levels of distress regarding sleep includes clock-watching, as this allows them to keep track of how much time they are lying awake and, thus, how much sleep they are (or are not) attaining. According to the cognitive model of insomnia, these actions exacerbate dysfunctional beliefs about sleep and perpetuate sleep disturbances [12]. The results of this current study illustrate that the deleterious effects of these behaviors may also extend to perceived EF as well. Specifically, as the effects of dysfunctional beliefs about sleep appeared to be subsumed in the variable of sleep quality (insomnia severity), the latter variable may have a more direct effect on one’s perceived EF than the former.

This notion also shapes clinical decisions in the form of treatment planning, as it encourages clinicians to prioritize treating insomnia symptoms rather than simply prescribing longer sleep durations. It provides evidence that treatments including CBT-I, sleep hygiene interventions, and even relaxation techniques that help address sleep quality may be more beneficial than aspiring for a specific sleep quantity. As college students have consistently been found to attain lower-than-recommended sleep quantity [2,3], a focus on sleep quality as opposed to sleep quantity may be more appealing and more feasible to this population, making it more likely for them to engage in such interventions and subsequently reap their benefits.

Finally, the attained results suggest that perceived inhibitory control and cognitive flexibility are more sensitive to insomnia severity, whereas perceived working memory abilities may be more resilient in this regard. This distinction may also be helpful in enacting more personalized interventions: college-aged patients perceiving themselves as struggling with impulsivity (i.e., inhibitory control) or cognitive rigidity may benefit from the sleep quality interventions identified above, while individuals perceiving themselves as struggling more with working memory (i.e., mental manipulation of stimuli, etc.) may benefit more from other interventions, such as those more-so aimed at self-compassion and self-efficacy. Providing these more tailored treatment plans to college-age individuals in a clinical setting will help ensure that their presented concerns are addressed in the most optimal way and will help bolster patient satisfaction and reduce distressed and perceived dysfunction within this population.

## Figures and Tables

**Table 1:** Demographic Information of the Participants.

**Race**	***n*** **(%)**
American Indian/Alaska Native	0 (0%)
Asian	3 (1.42%)
Black or African American	27 (12.73%)
Native Hawaiian or Other Pacific Islander	0 (0%)
White or European American	156 (73.58%)
Multi-racial	19 (8.96%)
Unknown or Other	4 (1.89%)
Prefer not to say	3 (1.42%)
**Ethnicity**	***n*** **(%)**
Hispanic/Latinx	25 (11.79%)
Not Hispanic/Latinx	187 (88.21%)
**Sex**	***n*** **(%)**
Female	162 (76.42%)
Male	49 (23.11%)
I’d prefer not to say	1 (0.47%)

The table includes the participants’ self-reported race (N = 212), ethnicity (N = 212) and biological sex (N = 212).

**Table 2:** Zero-Order Correlations for Measures of Sleep and EF (N = 212).

Variable	PSQI Sleep Quantity	ISI	DBAS-16	CNSVS	BRIEF-A
PSQI Sleep Quantity	1.0				
ISI	−0.43 **	1.0			
DBAS-16	0.20 **	0.13	1.0		
CNSVS	0.05	0.05	0.12	1.0	
BRIEF-A	−0.20 **	0.53 **	0.05	0.08	1.0

PSQI = Pittsburg Sleep Quality Index; ISI = Insomnia Severity Index; DBAS-16 = Dysfunctional Belief About Sleep Scale; CNSVS = CNS Vital Signs; BRIEF-A = Behavioral Rating Inventory of Executive Function—Adult Version. ** *p* < 0.01.

**Table 3:** Standardized Beta Weights for Regression Analyses of Sleep quantity, Insomnia Severity, Dysfunctional beliefs about sleep, Age, Sex, and Sleep quantity by Dysfunctional beliefs about sleep (Moderator) Predicting CNSVS and Objective Measures of EF Facets.

Outcome Variables	PSQI Sleep Quantity *β* (*SE*)	ISI Score *β* (*SE*)	DBAS Score *β* (*SE*)	Age *β* (*SE*)	Sex: I’d Prefer Not to Say *β* (*SE*)	Sex: Male *β* (*SE*)	Sleep Variable *X* DBAS Score β (*SE*)	*R*^2,^ F-Statistic
CNSVS Inhibition (Stroop Commission Errors)	−0.59 (1.02)		1.05 (1.56)	−5.08 ** (1.20)	11.19 (13.72)	−3.82 (2.23)	−0.06 (0.13)	*R*^2^ = 0.12, *F*(6, 204) = 4.83, *p* < 0.001
		0.11 (0.52)	0.06 (0.62)	−5.24 ** (1.23)	9.30 (14.35)	−3.59 (2.24)	0.02 (0.10)	*R*^2^ = 0.12, (*F*(6, 204) = 4.69, *p* < 0.001
CNSVS Cognitive Flexibility (Shifting Attention Correct Responses)	0.42 (1.14)		0.71 (1.75)	−0.73 (1.34)	4.46 (15.34)	−0.18 (2.49)	−0.03 (0.15)	*R*^2^ = 0.02, *F*(6, 204) = 0.53, *p =* 0.78
		0.11 (0.57)	0.48 (0.69)	−0.67 (1.37)	3.46 (16.01)	−0.28 (2.50)	−0.03 (0.11)	*R*^2^ = 0.02, (*F*(6, 204) = 0.52, *p =* 0.79
CNSVS Working Memory (4-Part CPT: Part 4 Correct Responses)	−0.22 (0.99)		−0.84 (1.53)	−1.56 (1.17)	13.12 (13.40)	0.12 (2.17)	0.08 (0.13)	*R*^2^ = 0.02, *F*(6, 204) = 0.61, *p =* 0.72
		0.62 (0.50)	0.93 (0.60)	−1.27 (1.20)	9.13 (13.94)	0.22 (2.18)	−0.13 (0.09)	*R*^2^ = 0.02, (*F*(6, 204) = 0.83, *p =* 0.55

*β* = standardized beta, *R*^2^ = coefficient of determination, *SE* = standard error; ** *p* < 0.01.

**Table 4:** Standardized Beta Weights for Regression Analyses of Sleep quantity, Insomnia Severity, Dysfunctional beliefs about sleep, Age, Sex, and Sleep quantity by Dysfunctional beliefs about sleep (Moderator) Predicting BRIEF-A (Subjective) EF Scores.

Outcome Variables	PSQI Sleep Quantity β (*SE*)	ISI Score β (*SE*)	DBAS Score β (*SE*)	Age β (*SE*)	Sex: I’d Prefer Not to Say β (*SE*)	Sex: Male β (*SE*)	Sleep Variable X DBAS Score β (*SE*)	*R*^2,^ F-Statistic
BRIEF-A Inhibition	0.94 (0.76)		4.90 ** (1.16)	0.19 (0.89)	11.12 (10.15)	−3.33 * (1.65)	−0.41 ** (0.10)	*R*^2^ = 0.14, *F*(6, 204) = 5.49, *p <* 0.001
		0.83 * (0.37)	−0.02 (0.44)	−0.35 (0.88)	−1.78 (10.21)	−2.60 (1.60)	−0.00 (0.07)	*R*^2^ = 0.20, *F*(6, 204) = 8.53, *p* < 0.001
BRIEF-A Shift	1.27 (0.83)		4.91 ** (1.27)	0.66 (0.98)	2.96 (11.16)	−5.03 ** (1.81)	−0.42 ** (0.11)	*R*^2^ = 0.13, *F*(6, 204) = 4.95, *p* < 0.001
		1.06 ** (0.40)	0.16 (0.48)	0.19 (0.96	−11.87 (11.23)	−4.30 * (1.75)	−0.05 (0.08)	*R*^2^ = 0.19, *F*(6, 204) = 7.90, *p* < 0.001
BRIEF-A Working Memory	1.06 (0.93)		6.53 ** (1.41)	−0.62 (1.09)	30.86 ** (12.42)	−6.11 ** (2.01)	−0.53 ** (0.12)	*R*^2^ = 0.21, *F*(6, 204) = 8.81, *p* < 0.001
		0.75 (0.45)	−0.25 (0.54)	−1.45 (1.07)	16.74 (12.53)	−5.18 ** (1.96)	0.06 (0.08)	*R*^2^ = 0.26, *F*(6, 204) = 11.83, *p* < 0.001

*β* = standardized beta, *R*^2^ = coefficient of determination, *SE* = standard error; * *p* < 0.05, ** *p* < 0.01.

## Data Availability

The raw data supporting the conclusions of this article will be made available by the authors on request.

## Appendix A

### Appendix A.1

**Table A1:** Adapted Table of Validity Criteria for CNSVS Tests.

Neurocognitive Test	Validity Criteria
Stroop Test	[Simple RT < (Complex RT Correct * 0.1) + Complex RT Correct] AND [Complex RT Correct < (Stroop RT Correct * 0.1) + Stroop RT Correct] AND (Complex Correct > Complex Errors) AND (Stroop Correct > Stroop Errors)
Shifting Attention Test (SAT)	SAT Correct Responses > SAT Errors
Four-Part Continuous Performance Test (FPCPT)	Part 2 Correct Responses > 2 AND Part 2 Correct Responses > Part 2 Incorrect Responses * AND Part 3 Correct Responses > 5 AND Part 3 Correct Responses > Part 3 Incorrect Responses * AND Part 4 Correct Responses > 5 AND Part 4 Correct Responses > Part 4 Incorrect Responses *

Note: FPCPT Part 2, 3, and 4 Commission Errors * are labeled as Incorrect Responses *.

### Appendix A.2

**Table A2:** Summary Statistics for Measures of Sleep and EF (N = 212).

Variable	M	SD
PSQI Sleep quantity	7.00	1.36
ISI	7.76	5.42
DBAS-16	4.55	5.06
CNSVS	92.97 (SS)	15.15
BRIEF-A	54.01 (T)	11.55
